# The route of infection with *Leptospira interrogans* serovar Copenhageni affects the kinetics of bacterial dissemination and kidney colonization

**DOI:** 10.1371/journal.pntd.0007950

**Published:** 2020-01-06

**Authors:** Nisha Nair, Mariana Soares Guedes, Catherine Werts, Maria Gomes-Solecki

**Affiliations:** 1 Department of Microbiology, Immunology and Biochemistry, The University of Tennessee Health Science Center, Memphis, Tennessee, United States of America; 2 Immuno Technologies, Inc, Memphis, Tennessee, United States of America; 3 Institut Pasteur, Biology and Genetics of the Bacterial Cell Wall Unit, Paris, France; University of Connecticut Health Center, UNITED STATES

## Abstract

The goal of this study was to characterize how natural routes of infection affect the kinetics of pathogenic Leptospira dissemination to blood and kidney. C3H/HeJ mice were sublethally infected with *L*. *interrogans* serovar Copenhageni FioCruz L1-130 (*Leptospira*) through exposure of a dermis wound and through oral and nasal mucosa, in comparison to uninfected mice and to mice infected via standard intraperitoneal inoculation. In striking contrast to oral mucosa inoculation, transdermal and nasal mucosa infections led to weight loss, renal colonization and inflammation, as previously observed for conjunctival and intraperitoneal infections. However, the timing at which *Leptospira* gained access to blood, as well as *Leptospira*’ colonization of the kidney and shedding in urine, differed from intraperitoneal infection. Furthermore, a comparative analysis of transcription of pro-inflammatory mediators in kidney and total immunoglobulin isotyping in serum from infected mice, showed increased innate immune response markers (KC, MIP-2, TNF-α) and lower Th1 associated IFN-γ in kidney, as well as lower Th1 associated IgG2a in mice infected through the nasal mucosa as compared to intraperitoneal infection. We conclude that the route of infection affects the timing at which *Leptospira* gains access to blood for dissemination, as well as the dynamics of colonization and inflammation of the kidney.

## Author summary

Leptospirosis is a neglected disease caused by pathogenic *Leptospira* that affects humans and animals. Hosts contract *Leptospira* after exposure to contaminated water through cuts and bruises on skin and mucous membranes. We hypothesized that the route of infection may affect the kinetics of *Leptospira* dissemination to tissues and leptospirosis progression. In this study, we evaluated the clinical outcomes, and kidney colonization and inflammation after exposure of mice to pathogenic *Leptospira* using three natural routes of infection. In contrast to transdermal and nasal mucosa, infection through oral mucosa did not cause weight loss and did not result in renal colonization or inflammation. We also found that different natural routes of infection affect the timing at which *Leptospira* gain access to blood for dissemination, as well as bacterial burden and levels of pro-inflammatory markers in kidney. Precise timing of bacterial dissemination in blood and urine are important differences to consider for assessment of clinical signs of leptospirosis and for development of diagnostic assays for direct detection of *Leptospira* in human and veterinary biological samples. These studies also provide disease model tools in which to test the efficacy of vaccine candidates using natural routes of infection.

## Introduction

Zoonotic diseases are a major concern to human health even in our era of medical and scientific advancement. Leptospirosis, caused by pathogenic *Leptospira spp*. is a neglected emerging zoonotic disease prevalent in industrialized urban, suburban, and rural regions, and is endemic to areas with tropical and temperate climate. Pathogenic *Leptospira spp*. infect a wide range of vertebrate animals as reservoir hosts, most of which are asymptomatic carriers. Rodents, specifically rats and mice, are carrier hosts that contaminate water and soil with their urine. Humans are considered incidental hosts that acquire infection after exposure to contaminated sources through abraded skin and mucous membranes or consumption of contaminated food [1]. Symptoms can range from asymptomatic to mild febrile illness culminating in multi-organ failure, if left untreated [2].

Exposure to *Leptospira* under natural conditions, i.e. entrance of *Leptospira* through skin and mucosa, was recently evaluated in rats and it was found that mucosal infection led to kidney colonization associated with higher excretion of *Leptospira* [3]. Work on mouse models of leptospirosis using adult mice suggested that serovar, inoculum dose and route of infection affected the kinetics of disease progression [4], [5], [6], [7], [8], [9], [10]. Others have observed the same association between inoculum dose and lethal leptospirosis in hamsters [11], [12]. The month-long lag between exposure and onset of symptoms among the Springfield Triathalon athletes [13], [2] compelled us to ask the question of how routes of infection affect the kinetics of leptospirosis and whether oro-nasal infection can be achieved in mice. In this study, we used the C3H-HeJ sublethal model of leptospirosis to determine how exposure to *L*. *interrogans* serovar Copenhageni FioCruz L1-130 through the transdermal and oro-nasal routes of infection affect the timing of bacterial dissemination to blood and urine as well as the associated clinical outcomes and kidney pathology. Data are discussed taking into consideration our previous findings using the other route of natural infection, the ocular conjunctiva, CJ [10].

## Materials and methods

### Bacterial strains

We used *Leptospira interrogans* serovar Copenhageni strain Fiocruz L1-130 (henceforth *Leptospira*), culture passage 2 after hamster infection, originally isolated from a patient in Brazil. *Leptospira* was cultured as previously described [10] and enumerated by dark-field microscopy (Zeiss USA, Hawthorne, NY) that was confirmed by qPCR (StepOne Plus, Life Technologies, Grand Island, NY).

### Animals

10-week old C3H/HeJ mice were purchased from The Jackson Laboratories (Bar Harbor, ME) and acclimatized for one week at the pathogen-free environment in the Laboratory Animal Care Unit of the University of Tennessee Health Science Center.

### Ethics statement

This study was carried out in accordance with the Guide for the Care and Use of Laboratory Animals of the NIH. The protocols were approved by the University of Tennessee Health Science Center (UTHSC) Institutional Animal Care and Use Committee, Animal Care Protocol Application, Permits Number 14–018 and 16–070.

### Infection of mice

Intraperitoneal infection was done as described previously using a dose of ~10^8^ virulent *Leptospira* in sterile PBS. Bacteria were counted in a Petroff-Hausser chamber under a dark field microscope and confirmed by qPCR. For transdermal infection, a wound was generated on the back of anesthetized mice. One square inch on the lower back was shaved and the exposed skin was scraped with a sterile razor just enough to create a superficial abrasion without bleeding. Subsequently, ~10^8^ spirochetes in 50–100 μl sterile PBS was applied on the transdermal wound and covered using an occlusive bandage. The protective bandage was removed the next day. For oral mucosa infection, 10^8^ spirochetes in 25 μl sterile PBS were deposited in the buccal cavity of anesthetized mice who swallowed the inoculum. For nasal mucosa infection, mice were anesthetized and a maximum of 20 μl of sterile PBS containing ~10^8^ spirochetes were deposited as small drops into each nostril, synchronized with inhalation. Oral and nasal mucosa experiments were done side by side. Groups of mice inoculated with endotoxin free PBS (Dulbecco) into the peritoneum (IP Ctrl), into the buccal cavity (OM Ctrl), into the nostrils (NM Ctrl) and deposited on the transdermal wound (TD Ctrl) were kept as negative controls. Body weights were monitored daily. Urine was also collected on a daily basis for 15 days post infection by gently massaging the bladder area and the urine was collected into sterile aluminum foil. Blood (up to 20 μl) was collected every other day by tail nick for 15 days. At termination, kidneys were collected for quantification and culture of spirochetes, and for quantification of inflammatory and fibrosis transcripts.

### ELISA

Concentration of total immunoglobulin IgM, IgG, IgG1, IgG2a and IgG3 was determined using Ready-Set-Go ELISA kits (eBioscience) in mouse serum. *Leptospira*-specific-IgM and -IgG antibodies were detected in serum using heat-killed *Leptospira* (56°C for 30min). The plates were coated with 100 μl of heat-killed *Leptospira* bacteria (4 mg/ml) in 100 mM sodium carbonate (pH 9.7).

### RT-PCR, and q-PCR

DNA was extracted per manufacturers’ instructions from urine, blood, and kidney using a NucleoSpin tissue kit (Clontech). Quantification of *Leptospira* 16s rRNA was done using TAMRA probe and primers from Eurofins (Huntsville, AL) by real-time PCR (qPCR) (StepOne Plus). RNeasy mini kit (Qiagen) was used to extract total RNA followed by reverse transcription using a high-capacity cDNA reverse transcription kit (Applied Biosystems). Real-time PCR on the cDNA was performed as described [9]. For RT-PCR, we used TAMRA probes specific for inducible nitric oxide synthase (iNOS), Collagen A1 (ColA1), keratinocyte-derived chemokine (KC, CxCL1), macrophage inflammatory protein 2 (MIP-2, CxCL2), RANTES (CCL5), tumor necrosis factor alpha (TNF-α) and interferon gamma (IFN-γ). β-actin was used as control for the comparative CT method [14].

### Kidney histopathology

Kidneys were excised from mice after euthanasia and fixed in 10% formalin. Presence of *Leptospira* in the kidney was determined by Warthin-Starry silver staining of kidney sections at termination. Formalin-fixed paraffin embedded tissues were stained with periodic acid-Schiff–Diastase (PAS-D). Stained sections were evaluated for interstitial inflammation, glomerular morphology and size using an Axio Zeiss Imager A1 light microscope. Slides were viewed in a blinded manner.

### Statistics

Two-tailed unpaired t-test with Welch’s correction was used to analyze differences between infected and non-infected groups in body weight and glomeruli size in kidney. Non-parametric unpaired Mann-Whitney Exact test was used to evaluate differences in *Leptospira* burden, cytokines and fibrosis mediators in kidney, and antibody in serum, between infected and non-infected groups. Ordinary One-Way ANOVA was used to compare IgG subtypes between infected groups. Statistical analysis was done using GraphPad Prism software, α = 0.05.

## Results

### Mice infected through the oral mucosa did not lose weight, in contrast to mice infected through the nasal mucosa and via transdermal abrasion

**Fig 1:**
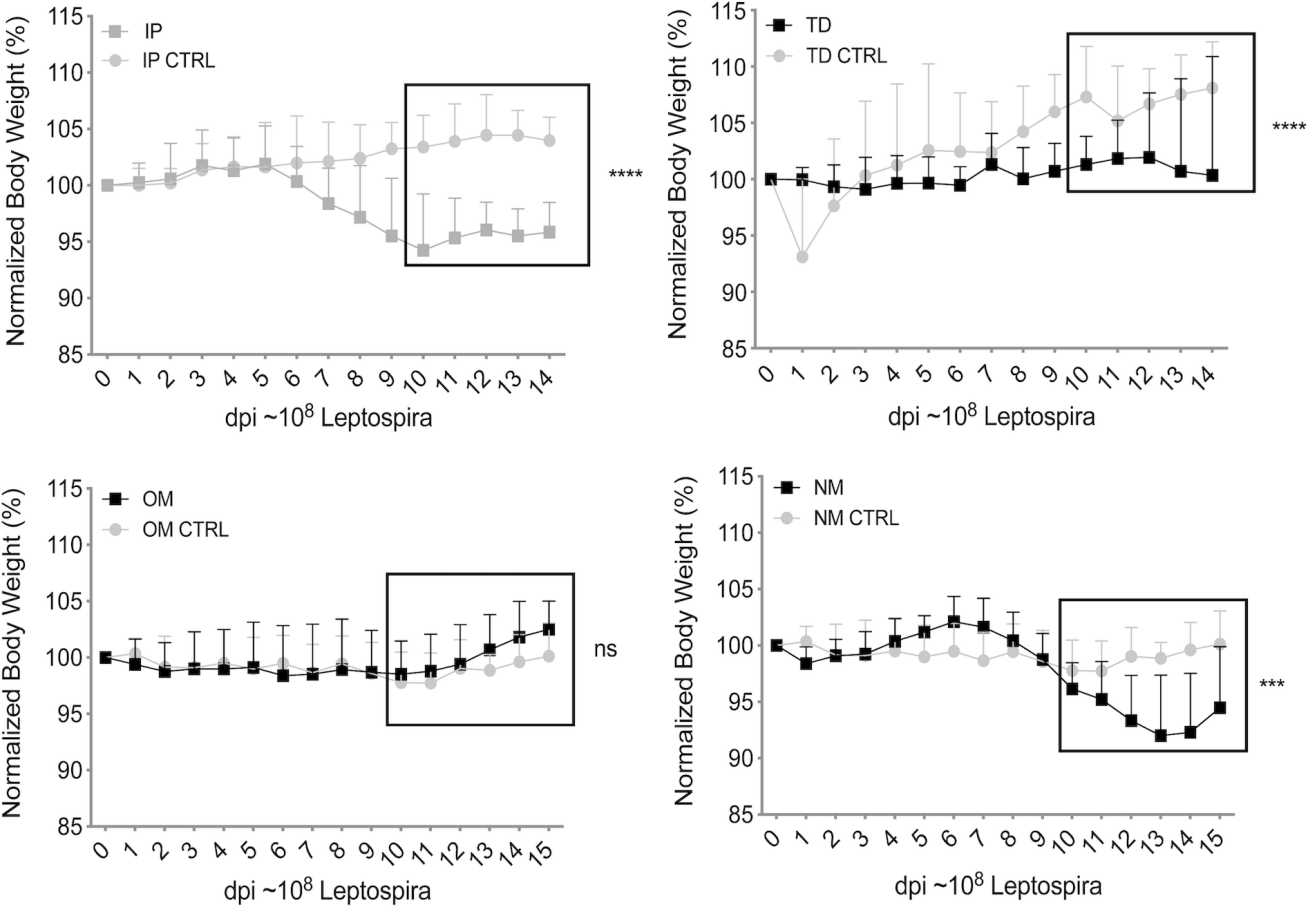
Weight loss after infection via three natural routes. Groups of C3H-HeJ mice were inoculated with an equivalent sublethal dose of *L*. *interrogans* serovar Copenhageni strain Fiocruz L1-130 (10^8^ culture P2 after hamster passage) via transdermal (TD), oral mucosa (OM), and nasal mucosa (NM) and were compared to uninfected controls and mice infected via the standard laboratory practice, intraperitoneal (IP). Body weights were recorded daily for two weeks post infection. Statistics by Unpaired t-test with Welch’s correction, infected versus uninfected control after day 10 post-infection: IP and TD p<0.0001, OM p = 0.1018 and NM p = 0.0002. *n* = 10–12 mice per group, representing two (OM, NM) or three (TD, IP) independent experiments.

10-week-old C3H/HeJ mice were infected with *Leptospira* by deposition of a ~10^8^ inoculum into the oral cavity (oral mucosa, OM), into the nares (nasal mucosa, NM) and on a transdermal abrasion (TD). Groups of mice inoculated with the same dose of *Leptospira* into the peritoneum (IP) were kept as positive controls. Groups of uninfected mice mock treated with PBS were used as negative controls. All infected mice across all groups survived until the end of the study, 15 days post infection. Weight records over 15 days post infection showed that mice infected via oral mucosa (OM) did not lose weight as compared to uninfected controls (p = 0.1018) (Fig 1). However, mice infected via nasal mucosa (NM) started losing a significant amount of weight on the second week of infection, on day 10 post-infection (p = 0.0002) as did IP infected mice, on days 7–8 post-infection (p<0.0001). Mice infected via transdermal abrasion did not gain nor did they lose weight, although the mice in the wound control group gained a significant amount of weight, p<0.0001 (Fig 1).

### The route of infection determines how fast pathogenic *Leptospira* gain access to blood for dissemination

**Fig 2:**
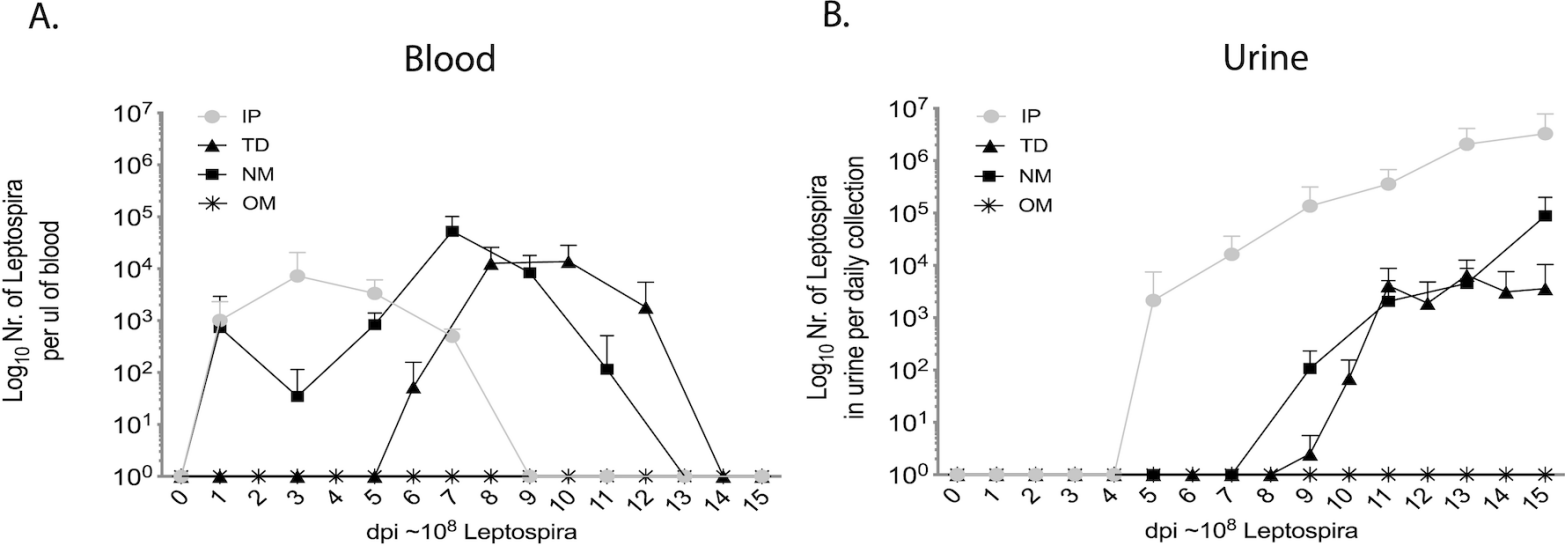
*Leptospira* dissemination in blood and urine. Groups of C3H-HeJ mice were inoculated with an equivalent sublethal dose of *L*. *interrogans* serovar Copenhageni strain Fiocruz L1-130 (10^8^) via three natural routes (transdermal (TD), oral mucosa (OM), nasal mucosa (NM)) compared to the standard intraperitoneal inoculation (IP). Bacterial burden in (A) blood and (B) urine was determined by *Leptospira* 16s rRNA qPCR. *n* = 10–12 mice per group, representing two (OM, NM) or three (TD, IP) independent experiments.

*Leptospira* was detected in blood on the first week on days 1, 3, 5, 7 post IP inoculation, on both weeks on days 1, 3, 5, 7, 9, 11 post nasal mucosa (NM) exposure, and on the second week on days 6, 8, 10, 12 after transdermal TD abrasion exposure (Fig 2A). No *Leptospira* was detected in blood after oral mucosa (OM) exposure. Thus, the timing at which *Leptospira* disseminated in blood was considerably different between the routes of infection. Furthermore, infection with 10^8^ *Leptospira* led to dissemination of equivalent numbers of *Leptospira* per μL of blood on respective peak days: ~1.3x10^4^ TD, ~5x10^4^ NM, ~7x10^3^ IP.

### Kidney colonization and shedding of *Leptospira* in urine

**Fig 3:**
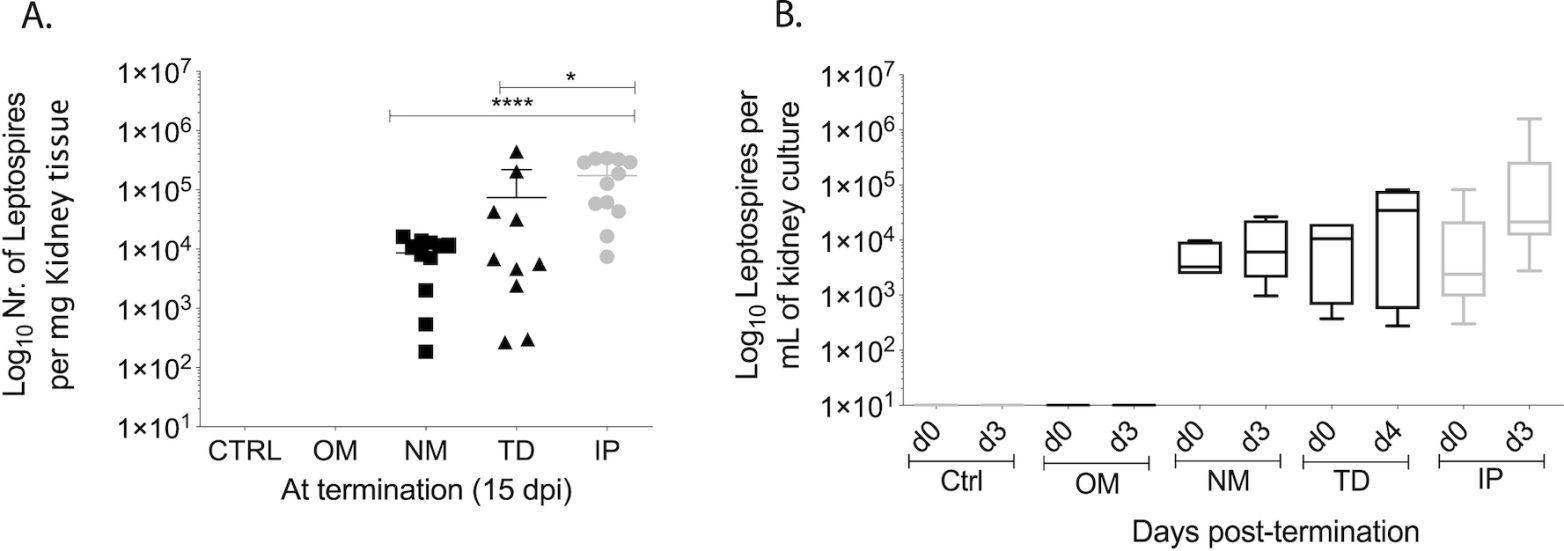
*Leptospira* burden and viability in kidney tissue. Groups of C3H-HeJ mice were inoculated with equivalent sublethal doses of *L*. *interrogans* serovar Copenhageni strain Fiocruz L1-130 (10^8^) via three natural routes, transdermal (TD), oral mucosa (OM), nasal mucosa (NM) and compared to uninfected controls (Ctrl) and to the standard intraperitoneal inoculation (IP). *Leptospira* burden and viability in kidney tissues was determined by qPCR of *Leptospira* 16s rRNA from (A) kidney tissue collected at termination on d15 post-infection and (B) from kidney culture. Statistics by unpaired Mann-Whitney Exact Test between NM, TD versus IP: **** p<0.0001; *p<0.05. A, *n* = 10–12 mice per group, data represents two (OM, NM) or three (TD, IP) independent experiments; B, *n* = 4 mice per group, representing one of 2–3 independent experiments.

Colonization of the kidney and shedding in urine was evaluated by qPCR and was observed on day 5 of the first week post infection via IP and on day 9 of the second week for both natural routes of infection, nasal NM and transdermal TD. No *Leptospira* was detected in urine of mice infected via oral mucosa (Fig 2B). After establishment of kidney colonization, mice ended up shedding a maximum of ~10^6^ *Leptospira* per μL of urine after IP, 6x10^3^ after TD and ~8.8x10^4^ after NM exposure on day 15 post infection (Fig 2B). At termination, 2 weeks post-infection, we detected ~1.7x10^5^ *Leptospira* per mg of kidney tissue in IP, ~7.4x10^4^ in TD, ~8.6x10^3^ in NM infected mice, whereas no spirochetes were detected in controls or in mice infected via the oral mucosa (OM) by qPCR (Fig 3A). *Leptospira* burden is summarized in Table 1 in comparison with previous CJ infection [10]. The viability of the spirochetes isolated from kidney was determined by qPCR quantification of the cultures kept for 3 to 4 days at 30°C in EMJH. Samples collected from cultures at d0, d3 or d4 post kidney culture showed increasing numbers of spirochetes for tissue collected from mice infected via IP, TD and NM but not for tissue harvested from controls or from mice infected via OM (Fig 3B). Presence of *Leptospira* in the kidney was also visualized by Warthin-Starry silver staining of IP and TD kidney sections at termination (S1 Fig). In the infected groups, spirochetes appeared as black colored aggregates in the tubules and as dispersed single cells interspersed through the renal tissue, which was absent in the uninfected control.

**Table 1:** *Leptospira* burden in blood, kidney, and urine of mice infected via IP, TD, NM, OM, and CJ [10] routes. The values shown are the average bacterial load observed on peak day for blood, and on termination day (d15) for kidney and urine.

	IP	TD	NM	OM	CJ [10]
μL Blood	7x10^3^	1.3x10^4^	5x10^4^	0	10^3^
mg Kidney	1.7x10^5^	7.4x10^4^	8.6x10^3^	0	10^4^
μL Urine	3.2x10^6^	6x10^3^	8.8x10^4^	0	10^6^

### Histopathological signs of inflammation in mice infected via IP and TD

Histopathology analysis of PAS-D stained paraffin embedded kidney tissue from IP and TD mice infected with equivalent inoculum doses showed increased mononuclear cell infiltration and the glomerular size was reduced by one-third as compared to controls (S1 Fig), as previously observed using a lower IP infectious dose in [9] and using another natural route of infection (CJ) with a comparable dose to our current study [10].

### Transcription of pro-inflammatory mediators and fibrosis markers in kidney

**Fig 4:**
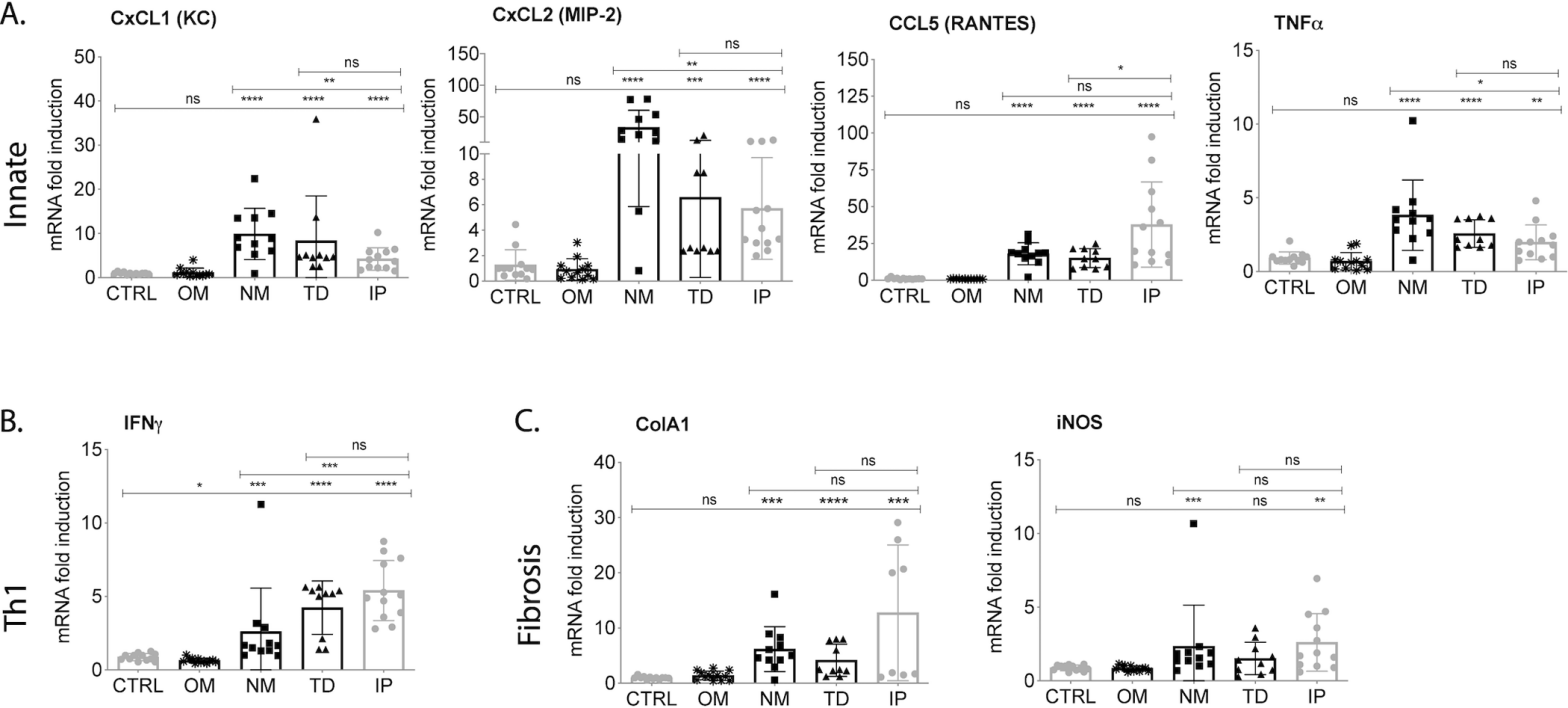
Transcription of pro-inflammatory and fibrosis markers in kidney of mice after inoculation with pathogenic *Leptospira*. C3H-HeJ mice were infected with equivalent sublethal doses of *L*. *interrogans* serovar Copenhageni strain Fiocruz L1-130 (10^8^) and kidney was collected two weeks post infection for quantitative PCR analysis of reverse-transcribed mRNA. (A) Innate response chemokines CxCL1, CxCL2 and CCL5 and cytokine TNF-α; (B) Th1 cytokine IFN-γ and (C) fibroblast activation marker collagen A1 (ColA1) and iNOS. Statistics by unpaired Mann-Whitney Exact Test between infected and uninfected control and between NM, TD versus IP: ns, not significant; **** p<0.0001; *** p<0.001; ** p<0.01, *p<0.05. *n* = 10–12 mice per group; Ctrl, control, OM, oral mucosa, NM, nasal mucosa, TD, transdermal and IP, intraperitoneal infections. Data represents two (OM, NM) or three (TD, IP) independent experiments.

Analysis of pro-inflammatory mRNA purified from kidney from mice infected via NM, TD and IP showed significant increases (p<0.01 to p<0.0001) of innate response chemokine and cytokine mediators (CxCL1/KC, CxCL2/MIP-2, CCL5/RANTES, TNF-α) and Th1 IFN-γ but not from mice infected via OM or from controls (Ctrl). The same was observed for analysis of a fibrosis mediator (the fibroblast activation marker collagen A1, ColA1) and an inflammation/fibrosis marker (the inducible nitric oxide, iNOS). Differences between infected NM, TD and IP versus OM and control mice are significant (Fig 4). KC, MIP-2 and TNF-α between infected mice were significantly higher in NM than IP (TD was not different), IFN-γ was significantly lower in NM than IP (TD was not different), and ColA1 and iNOS in NM and TD were not different than IP.

### B cell response

**Fig 5:**
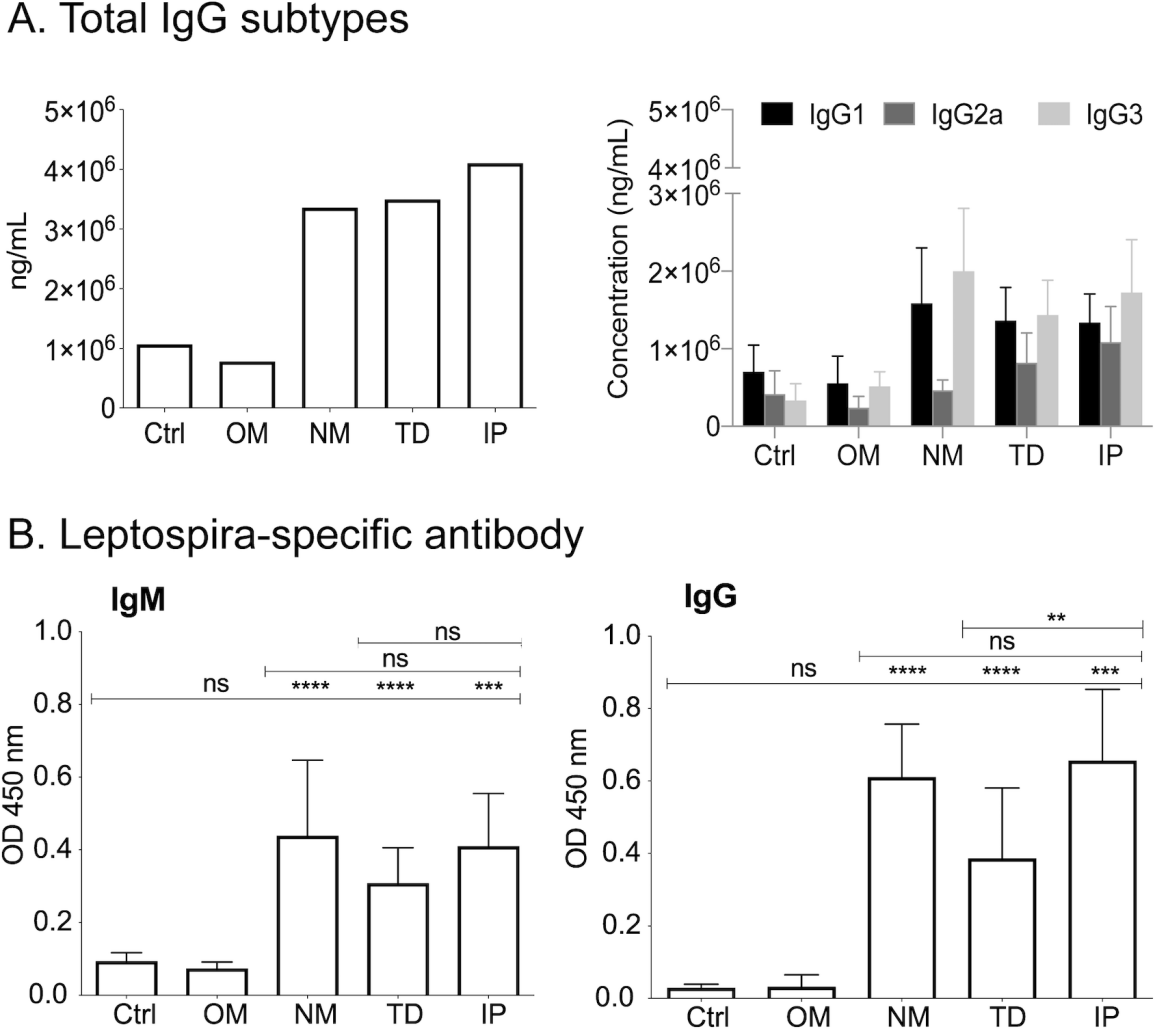
Antibody response in serum two weeks after infection. (A) Total IgG and isotypes and (B) *Leptospira*-specific IgM/IgG in serum from mice infected with 10^8^ *L*. *interrogans* serovar Copenhageni strain Fiocruz L1-130. Statistics by unpaired Mann-Whitney Exact Test between infected and uninfected control: ns, not significant, **** p<0.0001; *** p<0.001; ** p<0.01. *n* = 10–12 mice per group; Ctrl, control, OM, oral mucosa, NM, nasal mucosa, TD, transdermal and IP, intraperitoneal infections. Data represents two (OM, NM) or three (TD, IP) independent experiments.

NM, TD and IP infections led to a significant increase in the production of total immunoglobulin G (IgG) in serum compared to uninfected mice (Ctrl and OM): IP ~4098 μg/ml, TD ~3994 μg/ml, NM ~ 3357 μg/ml, OM ~778 μg/ml and Ctrl ~1065 μg/ml. Isotyping of total IgG in serum from mice infected by NM, TD and IP revealed a marked increase of IgG1 and IgG3 as compared to uninfected Ctrl and OM mice (Fig 5A). Interestingly, TD and IP infections also showed elevated IgG2a, although in lower amounts compared to IgG1 and IgG3, which contrasts with the NM infection that had considerably lower IgG2a. Ordinary one-way ANOVA analysis of IgG1, IgG2a and IgG3 between the infected groups (NM, TD and IP) shows that differences in IgG2a are statistically significant (*p* = 0.0013). We confirmed that increased concentrations of immunoglobulins were *Leptospira* specific by looking for IgM and IgG against heat-killed *Leptospira* (Fig 5B).

## Discussion

Animal models that allow for reproducible measurements of disease progression and pathology are essential for development of new therapies, vaccines and diagnostic assays for leptospirosis. In our previous studies, we chose to adapt the C3H-HeJ lethal model previously used by Pereira [15], Nally [4], and Vinetz [16] but rather than infecting young 4-week old mice, we infected mice at 10 weeks of age to allow enough time to include a 5-week vaccination scheme before challenge, considering that the murine immune system is functional after 5 weeks of age [17] and a typical immunization schedule requires at least 4 weeks. Application of the C3H-HeJ model as a possible correlate of sublethal human infection relies on identification of risk factors shared by mice and humans. Unlike the mouse TLR4 receptor, the TLR4 expressed in human immune cells does not recognize *Leptospira* LPS Lipid A [18, 19]. Therefore, compared to humans or hamsters, mice are more resistant to *Leptospira* infection [20] and usually don't die from acute leptospirosis [7]. However, C3H-HeJ mice also have impaired TLR4 sensing because they have a single amino acid substitution (aa712, P to H) within the coding region of the *tlr4* gene that makes this molecule hyporesponsive to bacterial LPS [21], including *Leptospira* LPS. Thus, impaired TLR4 sensing leads to a defective humoral response [22]; as a result, higher numbers of *Leptospira* persist in the host, cause disease that can be monitored though measurement of clinical scores, evade immune checkpoints and disseminate in blood to colonize the kidney; large amounts of *Leptospira* are then shed with urine [9] [10, 14].

It is important to note that mice can tolerate levels of LPS endotoxin 250 higher than humans [23] which makes them excellent reservoir hosts for a number of human pathogens, including *Leptospira*. For this reason, infection doses in mice have to be 2–3 logs higher than infectious doses in higher phylum vertebrates like humans. The condition *sine qua non* for a reservoir host is to be persistently infected with the pathogen it maintains in the enzootic cycle. Transmission to sylvatic rodents results in asymptomatic infection [24]. Thus, it is important to distinguish persistent infection that can result in symptomatic versus asymptomatic conditions.

Using a mouse strain with impaired TLR4 sensing (C3H-HeJ) and a relatively high dose of inoculum (10^6^ spirochetes), we developed a mouse model of persistent Leptospirosis in which we can measure empirical clinical signs of infection such as weight-loss and a number of other metrics that allow us to qualify and quantify differences in pathology in 10 week old mice [9]. However, effective vaccination strategies need to be confirmed after infectious challenge via natural enzootic transmission routes. When we tested the conjunctival (CJ) route of infection we found that a higher dose of *Leptospira* was necessary to produce bacterial dissemination and that the kinetics of dissemination appeared to differ from IP infection [10]. The aim of the present study was to use the C3H-HeJ mouse model and the infection dose (~10^8^) established previously for natural transmission via ocular conjunctiva [10] to evaluate if and how other possible natural routes of infection affect the timing of *Leptospira* dissemination through blood, colonization of the kidney, and shedding in urine in comparison to the standard laboratory route of infection (IP). One limitation of infection via a natural route is that we can’t precisely quantify the number of *Leptospira* that gain access to blood for dissemination. Although the inoculum dose used was equivalent between all routes, the number of spirochetes that breach the tissue and disseminate is contingent on the immune defense capability at each port of entry.

We found that weight differences between mice infected via oral mucosa were not significant from controls. However, differences in weight between infected and uninfected mice were significant after nasal mucosa and transdermal abrasion infections, as we had previously determined for infection through the ocular conjunctiva [10]. Our results show that infection through natural transmission routes such as nasal (NM), transdermal (TD) and conjunctival (CJ), but not oral mucosa (OM), lead to comparable disease that can be quantified by differences in weight loss.

*Leptospira* dissemination to blood happened within two weeks of infection and was considerably different after IP (d1-8), NM (d1-12) and TD (d6-14) infections than what we observed previously for CJ (d5-11, [10]). On the other hand, kidney colonization started in the second week of infection around days 7–9 (NM and TD, and CJ in [10]) and shedding in urine grew exponentially at that point, as observed previously [9], [10]. The burden of live *Leptospira* in kidney ranged between 10^4^ to 10^5^/ mg of tissue independently of natural route of infection (NM, TD, and CJ [10]) whereas kidney burden in IP infection was one Log higher. No *Leptospira* could be amplified from kidney tissue nor recovered by culture of kidney from mice infected via the oral mucosa or from controls. The burden of *Leptospira* shedding in urine and in kidney tissue was about two Logs lower in TD and NM than IP inoculations. This lower level of shedding and kidney burden could be reflective of a lower number of spirochetes being able to breach the immune defenses at each port of entry (NM and TD). Another difference was that although TD shedding reached a plateau on d12 when colonization appears to be established for this route, NM shedding remained in exponential growth and may have benefited from a longer infection schedule of 21 days to reach full colonization status. Our data shows a timing overlap between the two phases of *Leptospira* dissemination (blood dissemination and urine shedding) in mice infected via TD and NM routes of infection (this study) which is consistent with our observation when we used another natural route (CJ) of infection [10].

Lack of bacterial dissemination and colonization of the kidney after oral mucosa infection can’t be justified by lack of viability of the culture used for OM infection given that the same inoculum was used in parallel for OM and NM infections. One possible explanation for the lack of infection via oral mucosa in mice might be that *Leptospira* is neutralized by saliva and gastric acids as was shown after oral infection of hamsters [25]. Another explanation may be time of exposure to a liquid inoculum given that *Leptospira* provided over 10 days in drinking water caused infection [25]. Human-to-human infection is extremely rare but has occurred through sexual intercourse [26] and during lactation [27]. Our results don’t completely rule out the possibility that humans may acquire leptospirosis orally after prolonged consumption of contaminated food or water, but it may explain why those events are not reported often or that some sort of oral mucosa injury may mediate successful infection. Further studies to investigate the underlying mechanisms of efficient *Leptospira* killing in the oral cavity are needed.

Over the course of our studies we observed that the timing of establishment of colonization of the kidney in the second week of infection correlated well with weight loss for IP, NM, TD, and CJ but not for OM infection ([9], [10]). Thus, weight loss may be used to predict colonization of the kidney after infection.

Transcription of pro-inflammatory immune mediators (CxCL1/KC, CXCL2/MIP-2, CCL5/RANTES, TNF-α, IFN-γ and iNOS) and the fibrosis marker (ColA1) in the kidney were significantly more enriched in infected (NM, TD, IP) than uninfected mice (OM and controls). This was also consistent with our previous observations for CJ [10] infection using C3H-HeJ mice. Between infected mice (NM, TD and IP) we also observed significant differences in inflammatory markers. Of note was the increase in innate CxCL1, CxCL2 and TNF-α but not adaptive Th1 IFN-γ in NM infected mice. This could be explained by the fact that at termination, on d15 post-infection, mice infected through the NM route were still shedding *Leptospira* in urine exponentially, which could be driving an innate immune response pre full colonization. In previous studies we observed a good correlation between RNA transcription and protein expression of the chosen mediators in kidneys [22, 28].

B cell responses to *Leptospira* were measured by quantification and isotyping of total immunoglobulin (Ig) G, and *Leptospira*-specific IgM and IgG, in serum of uninfected and infected mice. Our results suggest that once *Leptospira* infection is established an immune response ensues that results in the classic initial production of IgM that switches to IgG by d15 post-infection. When we isotyped the total IgGs at d15 post-infection we found high IgG1 and IgG3 and lower IgG2a with significant differences in IgG2a between the routes of infection with lower IgG2a in NM infection. IgG3 is the first IgG to appear in serum as switching from IgM/D to IgG takes place and constitutes a minor proportion of IgG isotypes. It has modulating effector functions independent of T cell help such as complement dependent cytotoxicity and antibody-dependent cell cytotoxicity [29] [30]. The high concentration of IgG3 detected suggests that a T cell independent cytotoxic response to *Leptospira* is engaged early in the course of infection. As IgG2a is associated with Th1 responses our results suggest that in mice infected by nasal mucosa (NM) these effectors are not yet engaged by d15 post infection. This seems to be corroborated by the lower transcription of Th1 associated IFN-γ in kidney of NM infected mice.

Precise timing of bacterial dissemination in blood and urine are important differences to consider for assessment of clinical signs of leptospirosis and for development of diagnostic assays for direct detection of *Leptospira* in human and veterinary biological samples. These studies also provide disease model tools in which to test the efficacy of vaccine candidates using natural routes of infection.

## Supporting information

S1 Fig Renal colonization after transdermal infection. A, PAS-D staining showing mononuclear cell infiltration and reduced size of glomeruli in infected tissue and silver stain (Warthin-Starry) images showing *L*. *interrogans* serovar Copenhageni strain Fiocruz L1-130; the inset shows a magnified image of an aggregate of *Leptospira*; B, histogram depicting the difference in glomeruli size between infected and control groups and C, viability of *Leptospira* cultured from kidney tissue. P values by unpaired t test with Welch’s correction; *** p<0.001. Data represents one of three experiments.
(TIF)

## References

[1] Bharti AR, Nally JE, Ricaldi JN, Matthias MA, Diaz MM, Lovett MA, et al Leptospirosis: a zoonotic disease of global importance. Lancet Infect Dis. 2003;3(12):757–71. Epub 2003/12/04. 10.1016/s1473-3099(03)00830-2 . 14652202

[2] Haake DA, Levett PN. Leptospirosis in humans. Curr Top Microbiol Immunol. 2015;387:65–97. Epub 2014/11/13. 10.1007/978-3-662-45059-8_5 25388133 PMC4442676

[3] Zilber AL, Belli P, Grezel D, Artois M, Kodjo A, Djelouadji Z. Comparison of Mucosal, Subcutaneous and Intraperitoneal Routes of Rat Leptospira Infection. PLoS Negl Trop Dis. 2016;10(3):e0004569 Epub 2016/04/01. 10.1371/journal.pntd.0004569 27031867 PMC4816568

[4] Nally JE, Fishbein MC, Blanco DR, Lovett MA. Lethal infection of C3H/HeJ and C3H/SCID mice with an isolate of Leptospira interrogans serovar copenhageni. Infect Immun. 2005;73(10):7014–7. 10.1128/IAI.73.10.7014-7017.2005 16177383 PMC1230959

[5] Santos CS, Macedo JO, Bandeira M, Chagas-Junior AD, McBride AJ, McBride FW, et al Different outcomes of experimental leptospiral infection in mouse strains with distinct genotypes. J Med Microbiol. 2010;59(Pt 9):1101–6. Epub 2010/06/19. 10.1099/jmm.0.021089-0 . 20558584

[6] Bandeira M, Santos CS, de Azevedo EC, Soares LM, Macedo JO, Marchi S, et al Attenuated nephritis in inducible nitric oxide synthase knockout C57BL/6 mice and pulmonary hemorrhage in CB17 SCID and recombination activating gene 1 knockout C57BL/6 mice infected with Leptospira interrogans. Infect Immun. 2011;79(7):2936–40. Epub 2011/05/18. 10.1128/IAI.05099-11 21576342 PMC3191955

[7] Fanton d'Andon M, Quellard N, Fernandez B, Ratet G, Lacroix-Lamande S, Vandewalle A, et al Leptospira Interrogans induces fibrosis in the mouse kidney through Inos-dependent, TLR- and NLR-independent signaling pathways. PLoS Negl Trop Dis. 2014;8(1):e2664 10.1371/journal.pntd.0002664 24498450 PMC3907306

[8] Ratet G, Veyrier FJ, Fanton d'Andon M, Kammerscheit X, Nicola MA, Picardeau M, et al Live imaging of bioluminescent leptospira interrogans in mice reveals renal colonization as a stealth escape from the blood defenses and antibiotics. PLoS Negl Trop Dis. 2014;8(12):e3359 10.1371/journal.pntd.0003359 25474719 PMC4256284

[9] Richer L, Potula HH, Melo R, Vieira A, Gomes-Solecki M. Mouse model for sublethal Leptospira interrogans infection. Infect Immun. 2015;83(12):4693–700. 10.1128/IAI.01115-15 26416909 PMC4645400

[10] Sullivan JP, Nair N, Potula HH, Gomes-Solecki M. Eye-Drop Inoculation Leads to Sublethal Leptospirosis in Mice. Infect Immun. 2017 10.1128/IAI.01050-16 . 28115508 PMC5364295

[11] Haake DA. Hamster model of leptospirosis. Curr Protoc Microbiol. 2006;Chapter 12:Unit 12E 2 Epub 2008/09/05. 10.1002/9780471729259.mc12e02s02 18770576 PMC2667198

[12] Coutinho ML, Matsunaga J, Wang LC, de la Pena Moctezuma A, Lewis MS, Babbitt JT, et al Kinetics of Leptospira interrogans infection in hamsters after intradermal and subcutaneous challenge. PLoS Negl Trop Dis. 2014;8(11):e3307 Epub 2014/11/21. 10.1371/journal.pntd.0003307 25411782 PMC4239013

[13] Morgan J, Bornstein SL, Karpati AM, Bruce M, Bolin CA, Austin CC, et al Outbreak of leptospirosis among triathlon participants and community residents in Springfield, Illinois, 1998. Clin Infect Dis. 2002;34(12):1593–9. Epub 2002/05/29. 10.1086/340615 . 12032894

[14] Potula HH, Richer L, Werts C, Gomes-Solecki M. Pre-treatment with Lactobacillus plantarum prevents severe pathogenesis in mice infected with Leptospira interrogans and may be associated with recruitment of myeloid cells. PLoS Negl Trop Dis. 2017;11(8):e0005870 Epub 2017/08/26. 10.1371/journal.pntd.0005870 28841659 PMC5589268

[15] Pereira MM, Andrade J, Marchevsky RS, Ribeiro dos Santos R. Morphological characterization of lung and kidney lesions in C3H/HeJ mice infected with Leptospira interrogans serovar icterohaemorrhagiae: defect of CD4+ and CD8+ T-cells are prognosticators of the disease progression. Exp Toxicol Pathol. 1998;50(3):191–8. 10.1016/S0940-2993(98)80083-3 . 9681649

[16] Viriyakosol S, Matthias MA, Swancutt MA, Kirkland TN, Vinetz JM. Toll-like receptor 4 protects against lethal Leptospira interrogans serovar icterohaemorrhagiae infection and contributes to in vivo control of leptospiral burden. Infect Immun. 2006;74(2):887–95. 10.1128/IAI.74.2.887-895.2006 16428731 PMC1360355

[17] Landreth KS. Critical windows in development of the rodent immune system. Hum Exp Toxicol. 2002;21(9–10):493–8. Epub 2002/12/03. 10.1191/0960327102ht287oa . 12458906

[18] Nahori MA, Fournie-Amazouz E, Que-Gewirth NS, Balloy V, Chignard M, Raetz CR, et al Differential TLR recognition of leptospiral lipid A and lipopolysaccharide in murine and human cells. J Immunol. 2005;175(9):6022–31. 10.4049/jimmunol.175.9.6022 . 16237097

[19] Que-Gewirth NL, Ribeiro AA, Kalb SR, Cotter RJ, Bulach DM, Adler B, et al A methylated phosphate group and four amide-linked acyl chains in leptospira interrogans lipid A. The membrane anchor of an unusual lipopolysaccharide that activates TLR2. J Biol Chem. 2004;279(24):25420–9. 10.1074/jbc.M400598200 15044492 PMC2556802

[20] Gomes-Solecki M, Santecchia I, Werts C. Animal models of leptospirosis: of mice and hamsters. Frontiers in Immunology. 2017;under press. 10.3389/fimmu.2017.00058 PMC5318464 28270811

[21] Qureshi ST, Lariviere L, Leveque G, Clermont S, Moore KJ, Gros P, et al Endotoxin-tolerant mice have mutations in Toll-like receptor 4 (Tlr4). J Exp Med. 1999;189(4):615–25. Epub 1999/02/17. 10.1084/jem.189.4.615 9989976 PMC2192941

[22] Chassin C, Picardeau M, Goujon JM, Bourhy P, Quellard N, Darche S, et al TLR4- and TLR2-mediated B cell responses control the clearance of the bacterial pathogen, Leptospira interrogans. J Immunol. 2009;183(4):2669–77. 10.4049/jimmunol.0900506 . 19635914

[23] Copeland S, Warren HS, Lowry SF, Calvano SE, Remick D, Inflammation, et al Acute inflammatory response to endotoxin in mice and humans. Clin Diagn Lab Immunol. 2005;12(1):60–7. Epub 2005/01/12. 10.1128/CDLI.12.1.60-67.2005 15642986 PMC540200

[24] Ko AI, Goarant C, Picardeau M. Leptospira: the dawn of the molecular genetics era for an emerging zoonotic pathogen. Nat Rev Microbiol. 2009;7(10):736–47. Epub 2009/09/17. 10.1038/nrmicro2208 19756012 PMC3384523

[25] Asoh T, Saito M, Villanueva SY, Kanemaru T, Gloriani N, Yoshida S. Natural defense by saliva and mucosa against oral infection by Leptospira. Can J Microbiol. 2014;60(6):383–9. Epub 2014/05/28. 10.1139/cjm-2014-0016 . 24861456

[26] Harrison NA, Fitzgerald WR. Leptospirosis—can it be a sexually transmitted disease? Postgrad Med J. 1988;64(748):163–4. Epub 1988/02/01. 10.1136/pgmj.64.748.163 3174532 PMC2428802

[27] Bolin CA, Koellner P. Human-to-human transmission of Leptospira interrogans by milk. J Infect Dis. 1988;158(1):246–7. Epub 1988/07/01. 10.1093/infdis/158.1.246 . 3392418

[28] Lacroix-Lamande S, d'Andon MF, Michel E, Ratet G, Philpott DJ, Girardin SE, et al Downregulation of the Na/K-ATPase pump by leptospiral glycolipoprotein activates the NLRP3 inflammasome. J Immunol. 2012;188(6):2805–14. 10.4049/jimmunol.1101987 . 22323544

[29] Gavin AL, Barnes N, Dijstelbloem HM, Hogarth PM. Identification of the mouse IgG3 receptor: implications for antibody effector function at the interface between innate and adaptive immunity. J Immunol. 1998;160(1):20–3. Epub 1998/04/29. . 9551950

[30] Damelang T, Rogerson SJ, Kent SJ, Chung AW. Role of IgG3 in Infectious Diseases. Trends Immunol. 2019;40(3):197–211. Epub 2019/02/13. 10.1016/j.it.2019.01.005 . 30745265

