# Supplementary figures and images for: The route of infection with Leptospira interrogans serovar Copenhageni affects the kinetics of bacterial dissemination and kidney colonization
Source: PLoS Negl Trop Dis. 2020 Jan 6;14(1):e0007950. doi: 10.1371/journal.pntd.0007950 (PMC6964914; doi:10.1371/journal.pntd.0007950)

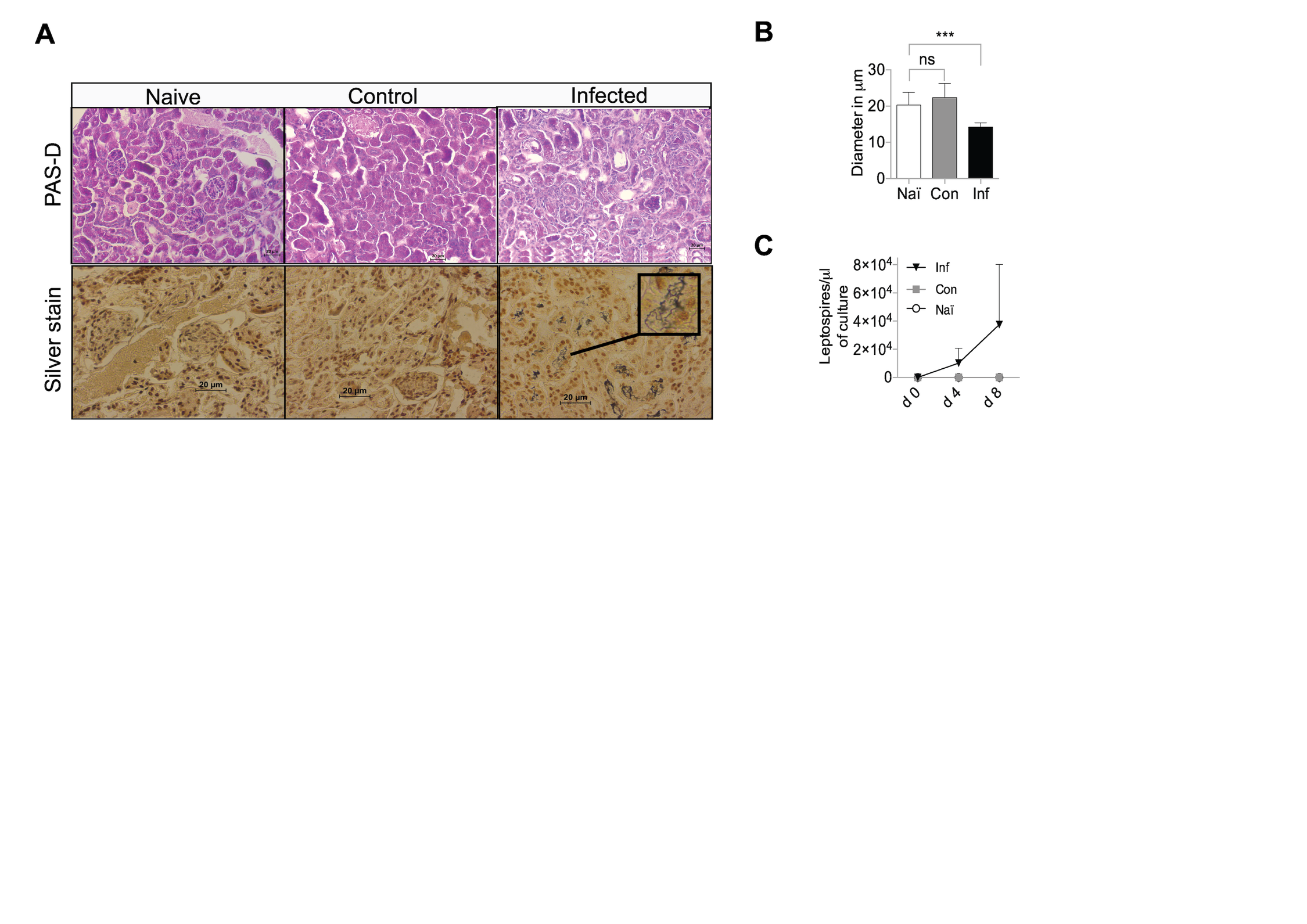

Supplement: S1 Fig — A, PAS-D staining showing mononuclear cell infiltration and reduced size of glomeruli in infected tissue and silver stain (Warthin-Starry) images showing L. interrogans serovar Copenhageni strain Fiocruz L1-130; the inset shows a magnified image of an aggregate of Leptospira; B, histogram depicting the difference in glomeruli size between infected and control groups and C, viability of Leptospira cultured from kidney tissue. P values by unpaired t test with Welch’s correction; *** p<0.001. Data represents one of three experiments. (TIF) [file pntd.0007950.s001.tif]
